# Ensuring the safety of newborns and children through community and healthcare actions

**DOI:** 10.1371/journal.pmed.1004730

**Published:** 2025-09-11

**Authors:** Moise Muzigaba, Ayda Taha, Mike English, Junior Mudji, Desire Habonimana, Sophie Jullien, Nuhu Yaqub

**Affiliations:** 1 World Health Organization, Headquarters, Geneva, Switzerland; 2 University of Oxford, Oxford, England,; 3 Hôpital Evangélique de Vanga, Vanga Mission, Kinshasa, Democratic Republic of the Congo; 4 Department of Family Medicine and Primary Care, Protestant University of Congo, Kinshasa, Democratic Republic of the Congo; 5 Centre de Recherche Universitaire en Santé, Department of Community Medicine, University of Burundi, Bujumbura, Burundi; 6 Centre for Tropical Medicine and Global Health, University of Oxford, Oxford, United Kingdom; 7 World Health Organization, Office for Quality of Care and Patient Safety, Regional Office for Europe, Athens, Greece

## Abstract

World Patient Safety Day 2025 is dedicated to “Safe Care for Every Newborn and Every Child”. In this Perspective, we highlight how parents, relatives, and the community, through observation and communication with healthcare workers, can become full partners in the safety of newborns and children, preventing harm at home, on the ward, and throughout the health system.

Preventable harm to newborns and children is probably far more widespread than is documented, especially in low- and middle-income countries (LMICs). A review of 32 hospital studies involving over 33,000 children found that the percentage of patients experiencing harm during their hospital stay varied widely [1]. The analysis showed that in regular children’s wards, hospitals in comparable settings could expect anywhere from 4% to 54% of children to face at least one preventable harmful event, and from 7% to 92% in intensive care units. Yet, there is evidence to show that even low levels of error, whether through omissions or commissions in care, can affect child health and development in both the short and long term [2]. Aligned with the theme of World Patient Safety Day 2025, this perspective emphasizes the often overlooked need for safer care for newborns and children.

At the grassroots level, community engagement is critical for building safe and responsive primary healthcare systems. Community members can take part in health system governance, including participation in facility or hospital boards, to help align services with local needs and build trust. Women’s groups using “find the problem, test a solution, check the result” cycles have also promoted timely care-seeking and support preventive practices at home. This approach involves families and local partners meeting to spot a priority risk, find the associated problem, and test a solution through small, low-cost changes. The results are then checked and discussed to assess what worked, and are adapted or scaled accordingly. In Asia and Africa, these groups have reduced newborn deaths by about one-third when at least one-third of pregnant women are involved [3].

Healthcare settings must do more to improve safety for newborns and children. In many LMICs, parents and family members are often the only people who continuously watch a sick newborn or child every minute, noticing changes in skin tone, a missed feed, or a drip that has stopped. Listening to them is therefore not a courtesy but a life‑saving safety measure. In a study that followed 383 families on two children’s wards in a high-income setting, 8.9% of parents spoke up about 37 safety problems. Doctors examined each report and found that 62% were genuine medical errors, and 30% of those errors were harmful to the child. Strikingly, more than 40% of the harmful errors never appeared in the medical record, showing that families can uncover some risks that might be overlooked by providers [4].

Healthcare managers and healthcare workers also have a role to play. For example, they use plain language and two-way bedside rounds that invite parents or family members into the daily discussion as real partners in planning care, identifying risks, and preventing harm. An example of such a strategy is the “Patient‑ and Family‑Centred I‑PASS” approach which involves a simple script that invites parents to help present the child’s overnight course, voice concerns first, and “read back” the day’s plan. Providers then hand the family a one‑page summary before leaving the room. In seven North American hospitals, this co‑produced approach cut preventable adverse events from 20.7 to 12.9 per 1,000 patient‑days (a 38% reduction) without lengthening rounds or hindering teaching [5]. In addition to engaging families, involving children through age and developmentally appropriate language, visual tools, and play-based approaches, can help them better understand their care, feel more at ease, and speak up when something doesn’t seem right.

Increasing recognition of safety concerns have also resulted in policy shifts at government levels. For example, the Ryan’s Rule in Queensland, Australia, is a consumer‑led escalation process that lets any hospital patient or their family call for an independent clinical review when they believe the patient is getting worse and staff are not responding [6]. Meanwhile, the recent introduction of Martha’s Rule in the United Kingdom, which provides a similar escalation pathway, provides a structured and psychologically safe way for families to raise the alarm, turning bedside worry into timely specialist action and embedding shared ownership of safety across the National Health Service in the United Kingdom [7].

We note that strategies that succeed in well‑resourced settings may not always transfer wholesale to low-resource settings where one overwhelmed nurse may not even be able to join a doctor’s round. In all settings, language or social hierarchies can also separate families from healthcare workers. Class, wealth, gender, and professional status shape who is and feels heard in the care process, especially in traditionally hierarchical cultures and institutions [8]. Real engagement therefore requires co‑design, testing, and local adaptation of strategies to address issues of power, not just pledges to follow evidence‑based guidelines or policies. Health systems therefore need to focus on feasible actions, including those targeting how health systems function, rather than issuing policies that frontline teams lack the capacity to implement.

Looking ahead, we propose a tiered approach that can be aligned with the local context to guide the scale-up of family and community engagement strategies to ensure safe care for newborns and children (Fig 1).

**Fig 1:**
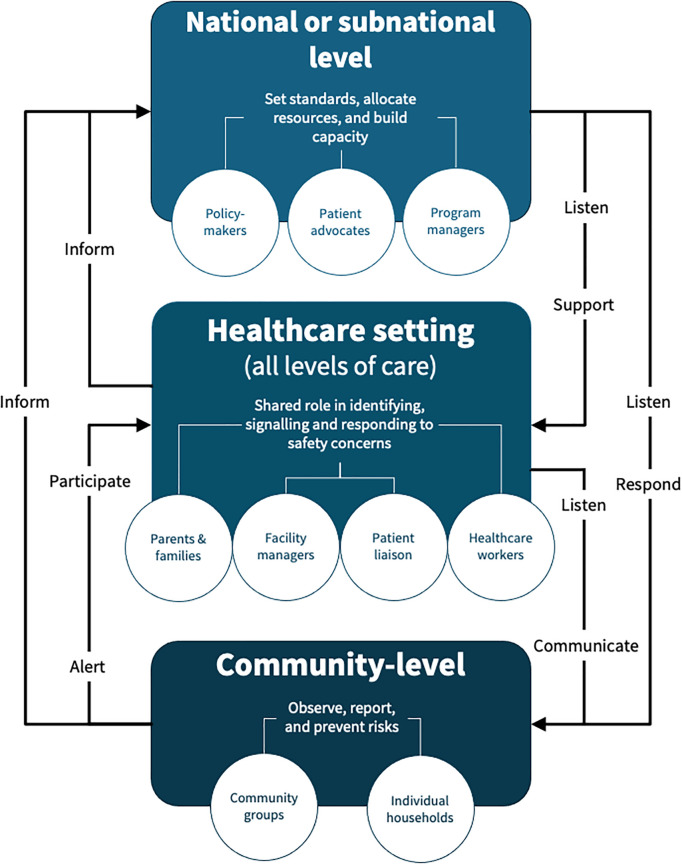
Safety action and feedback loop linking communities, parents, families, healthcare workers, managers, and policymakers to protect newborns and children. (Top) National and subnational levels can create the conditions that promote safety. (Middle) In healthcare settings, managers are key to operationalizing strategies and may need education and support to improve safety. (Bottom) At the community level, households, community members and groups can support prevention, promotion and timely action to enhance safety in partnership with primary healthcare team members.

At the community level, households, community members and groups can support prevention, promotion and timely action to improve safety in partnership with primary healthcare team members. Families and community health workers can observe newborns and children to raise alerts, promote care-seeking when they spot a danger, and improve local referral care systems. Existing or improved community forums can be used to develop an active dialogue with local and sub-national health system managers and keep policy makers at national levels informed of their challenges and experiences in healthcare and patient safety.

In healthcare settings, managers are key to operationalizing strategies. They can integrate family engagement into staff onboarding, routine team discussions such as safety huddles, incident reporting, quality improvement processes, and capacity building initiatives. Workflows and communication channels can be improved to empower parents and family members to raise concerns and for healthcare workers to respond. Managers can appoint patient representatives to participate effectively in facility management meetings. Healthcare providers can routinely involve caregivers in care decisions and daily care processes such as morning rounds, handovers, and discharge planning while continuing to coach parents on how to recognize danger signs in their newborn or child. They can post clearly worded escalation instructions at bedsides and in waiting areas, and encourage families to speak up or request urgent review if they are concerned.

At national and subnational levels, conditions that promote safety can be fostered. For example, governments can institutionalize parent, family member, and community engagement by embedding it into hospital licensing and accreditation requirements and quality of care standards. Financing mechanisms, including reimbursements, can be linked to safety goals, and systems mandating public reporting of family‑reported safety incidents can be established. Tools to enable families or communities to escalate safety concerns can be developed. Performance dashboards and incident reviews must also give equal weight to community and family‑reported data. Health workers will need better working conditions (including addressing workforce deficits) and support through structured training to improve their listening, communication, and partnership skills often degraded by burnout.

The World Patient Safety Day 2025, a global public health day observed on 17 September, is dedicated to “Safe Care for Every Newborn and Every Child” with the slogan “Patient safety from the start” [9]. Our proposed approach aligns with this event and fits squarely with several global health commitments. The Patient Safety Rights Charter from the World Health Organization (WHO) states that every person has the right to take part in decisions about their care (Right Ten) [10]. The Global Patient Safety Action Plan 2021–2030 also calls for engaging families in care decisions as well as system governance, service design, and safety oversight [11]. This is reflected in one of the plan’s core indicators, which measures whether a patient representative has been appointed to the governing board in at least 60% of hospitals [12]. Ensuring safe care for every newborn and child will require not only action from health systems and governments but also active participation from parents, families, and communities whose voices and vigilance must be recognized as essential to preventing harm and saving lives.

## Abbreviations

**:**
LMICs: low- and middle-income countries
WHO: World Health Organization
